# Direct evidence of flat band voltage shift for TiN/LaO or ZrO/SiO_2_ stack structure via work function depth profiling

**DOI:** 10.1038/srep43561

**Published:** 2017-03-02

**Authors:** Sung Heo, Hyoungsun Park, Dong-Su Ko, Yong Su Kim, Yong Koo Kyoung, Hyung-Ik Lee, Eunae Cho, Hyo Sug Lee, Gyung-Su Park, Jai Kwang Shin, Dongjin Lee, Jieun Lee, Kyoungho Jung, Moonyoung Jeong, Satoru Yamada, Hee Jae Kang, Byoung-Deog Choi

**Affiliations:** 1 Platform Technology Lab, Samsung Advanced Institute of Technology, 130, Samsung-ro, Yeongtong-gu, Suwon-si, Gyeonggi-do 16678, South Korea; 2 College of Information and Communication Engineering, Sungkyunkwan University, 2066 Seobu-ro, Jangan-gu, Suwon-si, Gyeonggi-do 16419, South Korea; 3 DRAM Technology Development Team, Semiconductor R&D Center, 1, Samsungjeonja-ro, Hwaseong-si, Gyeonggi-do 18448, South Korea; 4 Department of Physics, Chungbuk National University, 1, Chungdae-ro, Seowon-gu, Cheongju-si, Chungcheongbuk-do 28644, South Korea

## Abstract

We demonstrated that a flat band voltage (*V*_*FB*_) shift could be controlled in TiN/(LaO or ZrO)/SiO_2_ stack structures. The V_FB_ shift described in term of metal diffusion into the TiN film and silicate formation in the inserted (LaO or ZrO)/SiO_2_ interface layer. The metal doping and silicate formation confirmed by using transmission electron microscopy (TEM) and energy dispersive spectroscopy (EDS) line profiling, respectively. The direct work function measurement technique allowed us to make direct estimate of a variety of flat band voltages (*V*_*FB*_). As a function of composition ratio of La or Zr to Ti in the region of a TiN/(LaO or ZrO)/SiO_2_/Si stack, direct work function modulation driven by La and Zr doping was confirmed with the work functions obtained from the cutoff value of secondary electron emission by auger electron spectroscopy (AES). We also suggested an analytical method to determine the interface dipole via work function depth profiling.

Aggressive scaling down of the metal-oxide-semiconductor-field-effect-transistor (MOSFET) makes it possible to replace the conventional poly-silicon gate by a metal gate stack. The advantages of metal gates are low resistivity, a lack of charge depletion, and no need for doping in poly-Si, which can penetrate into the gate dielectric^1^. The ability to adjust threshold voltage control for low voltage operation in metal gate devices via the work function of the gate materials remains a key factor^2^. Among various metal gate candidate materials, TiN has been widely used because it is chemically inert, has a low resistivity, and is compatible with current processing techniques^3^. In general, the work function of the integrated metal-gate electrode/oxide/Si-sub structure is different from that of pure materials due to the formation of interfacial layers between each material. This phenomenon results in an effective work function (EWF), which is one of the most important parameters utilized in determining device performance^4^. The EWF can change by varying the gate-electrode and oxide interface properties. Therefore, work function engineering is required to control the EWF by handling the following conditions: (1) formation of the interface bonding, (2) charge movement in the interface (i.e. formation of interface dipole), and (3) generation of defects in the interface. The EWF reported to be increase by inserting LaO/HfSiO, AlO/HfLaSiO_x_, or HfO as a work function control layer because of the silicate layer formation between SiO_2_ and each work function control layer^5^,^6^,^7^. Z. Essa *et al*. reported^5^ the negative EWF shift caused by formation of a La-silicate layer in TiN/LaO/HfSiON/SiO_2_/Si-sub devices. A. Spessot *et al*. alternatively reported^8^ that the La-silicate caused a positive EWF shift. These results have been highly controversial.

In this study, the work function depth profiling technique has been applied to measure the work function of the gate electrodes directly; further, the work function was obtained from the cutoff energy of secondary electron energy distribution induced with the incident low energy (<2 keV) electron beam during Ar^+^ ion sputtering^9^,^10^. The advantage of this technique is to measure the work function of a localized area using a focused electron beam and measure an accurate work function of the surface using a low energy electron beam. These results demonstrated the mechanism of the work function changes, which were observed by using the LaO or ZrO as a work function control layer in TiN metal gate stacks.

## Experimental

We evaluated the V_FB_ shift by calculating the EWFs for the TiN/(LaO or ZrO)/SiO_2_ stack structures. A 70 Å thick thermal oxide was grown on a p-type Si(100) wafer, and the inserted layers (LaO, ZrO) were deposited using atomic layer deposition (ALD) with precursors of La(iPrCp)_3_ and ZrCl_4_, an oxidant (H_2_O, O_3_), and purging gas (Ar) in the temperature range of 300 °C to 350 °C. ALD cycles performed using alternating pulses (0.05 s) of precursors and oxidant. Purging gas was introduced between the pulses. Each cycle a layer <0.5 Å was generated. A TiN electrode was deposited by using chemical vapor deposition (CVD) between 600 and 700 °C at a pressure of 2 Torr at a precursor temperature of 55 °C and Ar carrier gas of 80 sccm. The samples were post-annealed by using a rapid thermal annealing (RTA) process at 1000 °C for 120 sec. The thickness of LaO and ZrO as the insert layers were 4.5, 7.0, 10.0 Å, and 5.0, 8.0, 11.0 Å, respectively. We measured the shift of the flat band voltage (V_FB_) via capacitance voltage measurements using a Boonton capacitance meter at 1 MHz. The film composition was determined using Rutherford backscattering (RBS). The RBS analysis was carried out with a 450 keV He^+^ probe beam, with an incident angle of 50° and a scattering angle of 70.5°. To analyze the cross-sectional composition and image the TiN/LaO/SiO_2_/Si stack, high-resolution transmission electron microscopy analysis was used with a Cs-corrected FEI Titan microscope operating at 300 kV, and TEM- energy dispersive spectroscopy (EDS) was used. PHI4700 Auger electron spectroscopy (AES) was used to directly measure the work function from cutoff energy of secondary electron energy distribution induced by the 1 keV primary electron beam. The sample bias was set at −30 V. The depth profiling of the work function was obtained during the 1 keV Ar^+^ ion sputtering. The work function measurements were also performed via ultraviolet photoemission spectroscopy (UPS) using a PHI 5000 Versaprobe (ULVAC-PHI) with a He I discharge lamp (*hν* = 21.2 eV).

The *V*_*FB*_ of the high dielectric gate oxide material stack structure such as the metal/high-k/SiO_2_/Si structure is given as follows^11^.


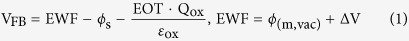


where *ϕ*_(m,vac)_ is the gate stack work function in vacuum, ∆V is the interfacial dipoles, *ϕ*_s_ is the silicon work function, EOT is the equivalent oxide thickness, *ɛ*_ox_ the permittivity of SiO_2_, and Q_ox_ is the equivalent oxide charge density, which is assumed to be located at the dielectric/silicon interface.

## Results and Discussion

**Figure 1:**
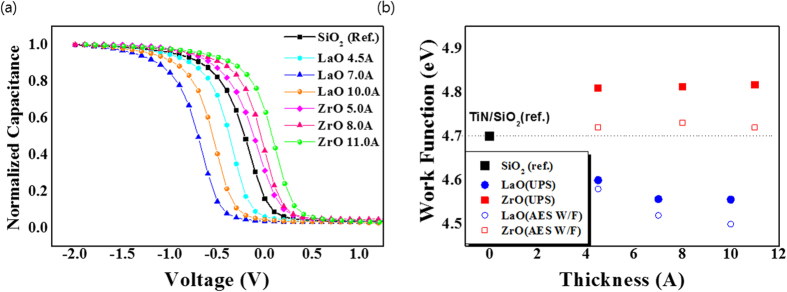
(**a**) Capacitance and voltage characteristics of LaO and ZrO at various deposition thicknesses and (**b**) the work function measurements observed using UPS and AES.

Figure 1(a) illustrates the *C-V* plot as a function of the thickness of LaO and ZrO. It can be noted that for LaO thicknesses up to 7 Å the insertion of a LaO layer induces a negative shift of *V*_*FB*_ with respect to the reference structure (TiN/SiO_2_/Si), which increases with the thickness. However, for a LaO thickness of 10 Å, a decrease of the negative shift of *V*_*FB*_ is observed, with a *V*_*FB*_ value below that corresponding to 7 Å. Our previous paper reported^12^ that the EWF exponentially decreased as La doping concentration increased in a TiN gate electrode. Accordingly, the chemical compositions of the TiN/LaO/SiO_2_/Si-sub stacks were measured via TEM-EDX and TEM, in order to interpret the above-mentioned results. When we introduced the LaO capping layer, *V*_*FB*_ increased (Fig. 1(a)), and the negative *V*_*FB*_ increased with increasing LaO thickness. Therefore, This imply that the threshold voltage (*V*_*th*_) can be adjusted by using a LaO layer in TiN/SiO_2_/Si stack structures. The maximum negative shift of *V*_*FB*_ was observed at the LaO capping layer of thickness 7 Å; however, the negative shift reduced when the thickness of the LaO capping layer exceeded 10 Å. In contrast to La, the *V*_*FB*_ of both materials shifted positively and exhibited a greater positive shift as the thickness of ZrO increased. The Zr-silicate showing a positive EWF was observed at the ZrO/SiO_2_ interface with a ZrO capping layer.

Figure 1(b) shows the work function at the TiN surface, measured via UPS and AES. The work function obtained from the AES method is smaller than that of the UPS method. However, the tendency to increase and decrease of work function obtained from both methods is in good agreement. In the case of the samples with inserted LaO layers, the work function from both techniques decreased as the LaO thickness increased up to 7 Å, while in the case of ZrO, the work function increased as the thickness of the ZrO increased.

These results were consistent with the results obtained from the *C-V* measurements.

**Figure 2:**
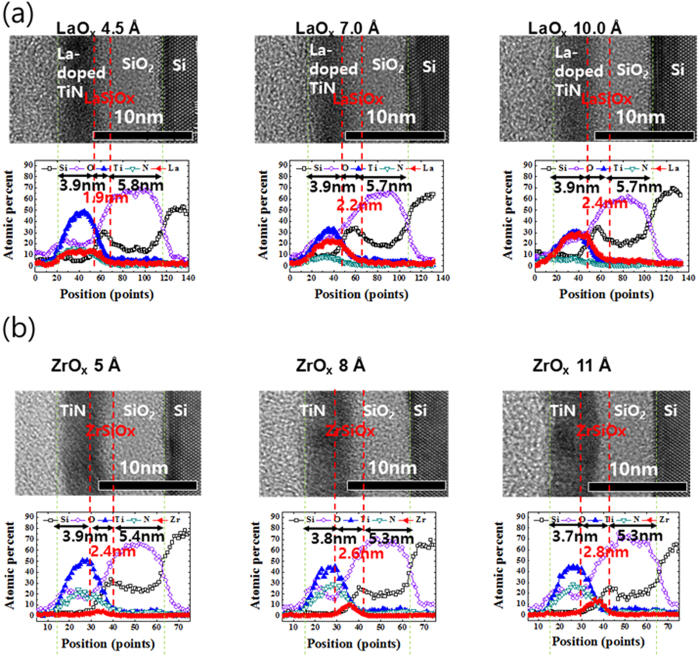
The cross-sectional TEM and TEM EDX spectrum of (**a**) LaO and (**b**) ZrO according to deposition thickness in a TiN/LaO, ZrO/SiO_2_/stack structure.

Figure 2(a) illustrates the TEM and TEM- EDS images of TiN/LaO/SiO_2_/Si-sub stacks of each sample. The spectra in Fig. 2(a) show that the La diffused into the entire TiN layer uniformly. As the thickness of LaO increased, the concentration of La increased; consequently, Si atoms piled up at the LaO/SiO_2_ interface, indicating that a La-silicate layer has formed at the interface. The diffusion of Si to the uppermost surface of the oxide layers has been reported by several groups^13^,^14^,^15^. The driving force of the emission of Si from the Si interfaces is believed to be the compressive stress at the SiO_2_/Si interface that occurs during oxidation^16^. As the thickness of LaO increased, the concentration of La in SiO_2_ also increased, indicating that La-silicate has formed at the interface. Based on simulations, A. Spessot *et al*.^8^ reported that EWF shifts positively as the La-silicate increases.

Figure 2(b) illustrates the TEM and TEM-EDS results of the TiN(3 nm)/ZrO/SiO_2_ (7 nm) stack structures post annealing at 950 °C. Contrary to the LaO capping layer, Zr was not observed in the TiN layer, although a Zr-silicate was formed at the interface of the ZrO and the lower SiO_2_. The concentration of Zr in the Zr-silicate increased with increasing the thickness of ZrO (5, 8, and 11 Å). The Zr-silicate was formed owing to the diffusion of Zr into SiO_2_, which results in a positive shift in the EWF. The amount of Zr in Zr-silicate is proportional to the positive shift of the EWF.

Table 1 showed the composition results obtained from RBS analysis of the TiN/LaO/SiO_2_/Si-sub stack with varied sample thicknesses. When the thickness of LaO was increased from 4.5 to 7 Å, and from 7 to 10 Å, the concentration of La in the TiN layer also increased from 3.1% to 5 at%, and from 5% to 6.8 at%, respectively.; the La concentration in the La-silicate layer increased from 1.3% to 1.5%, and from 1.5% to 1.8%, respectively. As a result, the EWF was minimized when the concentration of La was approximately 5%, which corresponds to the LaO thickness of 7 Å in Fig. 1(a). In our previous study^12^, density functional theory (DFT) calculations demonstrated that the EWF decreases exponentially as the concentration of La increases in a TiN gate electrode. On the other hand, the EWF increases linearly when the concentration of La in the interface between LaO and SiO_2_ increases. Experimentally, we could control the atomic concentration of La by varying the thickness of inserted-LaO layer.

If the atomic concentration of La exceeds 5%, a negative EWF shift obtained by further La doping in the TiN layer become saturated, whereas the increase in La-silicate caused the EWF to shift positively, resulting in an increase in the total EWF.

We suggest the following semi-empirical EWF equation for the TiN/LaO/SiO_2_/Si-sub stack to illustrate the above mentioned phenomenon:


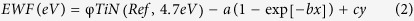


The work function of TiN is 4.7 eV, and the total EWF may be calculate from Eq. (2). In this case, a = 1.65, b = 0.74, and c = 0.98 are constants extracted from the quantitative RBS simulation, and x and y are the concentration (atomic%) of La in TiN and La-silicate, respectively.

**Figure 3:**
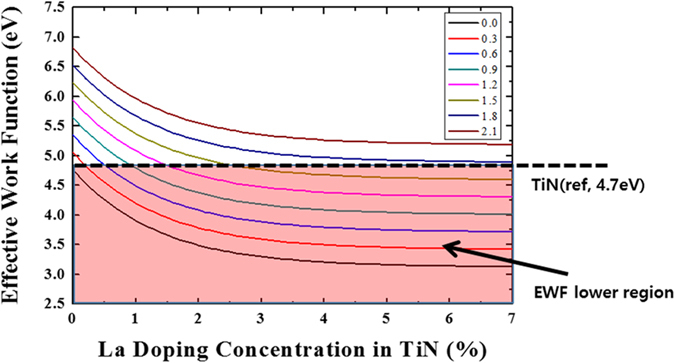
Calculated EWF as a function of La doping concentration in TiN and La-silicate (the inserted values) obtained from Equation (2).

Figure 3 shows the EWF as a function of La doping concentration in TiN, based on Eq. (2). The area marked using a red box represents the condition in which the EWF of the TiN/LaO/SiO_2_/Si-sub stack is lower than the work function of TiN(4.7 eV). These results are useful for adjusting the EWF in TiN/LaO/SiO_2_/Si-sub structured devices. We performed additional experiments using the inserted ZrO layer, which can be utilized as a work function control layer.

Table 2 showed the RBS quantitative analysis of the TiN/ZrO/SiO_2_/Si-sub stack for various sample thicknesses. It was estimated that little Zr exists in the TiN layer; however, the concentration of Zr in the Zr-silicate layer was increased from 5.7% to 11.2 and from 11.2% to 18.8 at% as the ZrO thickness was increased from 5 to 8 Å, and from 8 to 11 Å, respectively.

**Figure 4:**
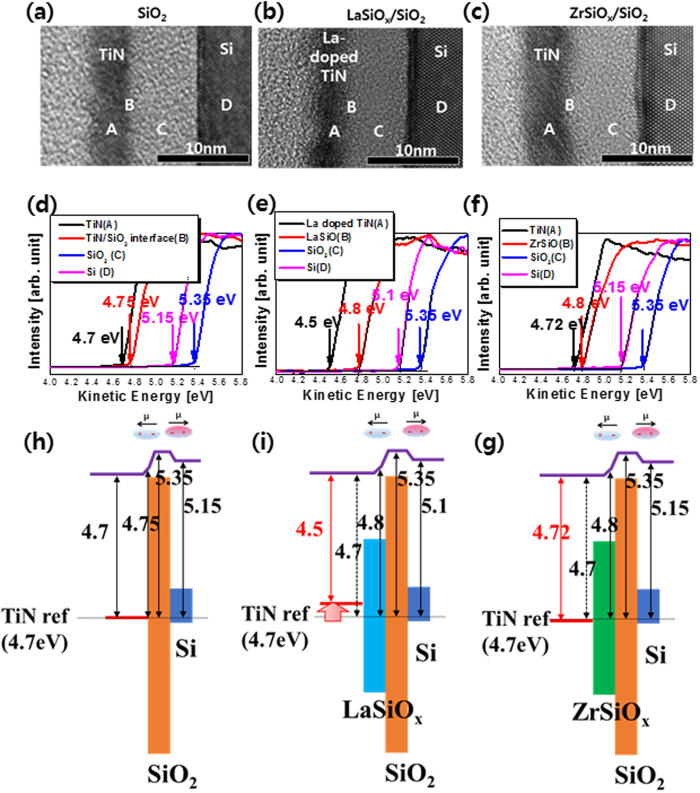
TEM image of (**a**) TiN/SiO_2_/Si (Ref.), (**b**) TiN/LaO 10 Å/SiO_2_/Si and (**c**) TiN/ZrO 11 Å/SiO_2_/Si stack structure. The work function of (**d**) reference sample (**e**) LaO and (**f**) ZrO sample according to the depth profiling. The schematic work function depth profiling of (**g**) reference sample, (**h**) LaO and (**i**) ZrO.

Figure 4 shows the work function depth profiling data of a TiN/(LaO or ZrO)/SiO_2_/Si stack structure.

Figure 4(a,b and c) show TEM images of a TiN/(without insert layer; reference TiN, LaO 10 Å, ZrO 11 Å)/SiO_2_/Si stack structure. Figure 4(d,e and f) show the work functions of TiN/(without insert layer; reference TiN, LaO 10 Å, ZrO 11 Å)/SiO_2_/Si stack samples, which were obtained from the cutoff energy of the secondary electron emission generated with the electron gun of Auger system^12^. Their Fermi levels (*E*_*F*_) were aligned when both the samples and analyzer were grounded. The hemispherical energy analyzer was used to obtain the secondary electron energy distribution emitted onto the sample by the incident electron beam. Therefore, the cutoff values, *E*_*on*_, of a secondary electron emission spectrum excited by the primary electron beam is the difference between vacuum levels (*E*_*vac*_) of the sample and energy analyzer as shown as follows,


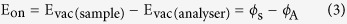


where φ_S_ and φ_A_ are the work functions of the sample and the analyzer, respectively.

Thus, *E*_*on*_ measures the difference between the work functions of the sample and the analyzer. The work function of the analyzer can be obtained by using samples with well-known work functions^9^,^17^. Using the work function measurement in AES system, the work function of the top surface in reference sample (without insert layer) and La doped TiN of LaO (10 Å) were determined to be 4.7 eV, and 4.5 eV, respectively.

Furthermore, the work function of the top surface (TiN) of the ZrO (11 Å) sample was measured to be 4.72 eV. The Zr atoms from the ZrO did not diffuse into the TiN, and the work function value was approximately 4.72 eV in these samples with the TiN as a reference. The La atoms in LaO were found to be diffused into the TiN layer, and the work function in these samples was measured to be 4.5 eV, which is low compared to that of the inserted ZrO layer. When compared to the RBS data shown Table 1, the quantified La doping concentration in LaO is 10 Å, which is measured at a concentration of approximately 5% La in the TiN layer. The work function of TiN reduced to 0.2 eV at samples containing concentration of 5% La.

The schematic diagrams of work function depth profiling for the reference sample (TiN/SiO_2_/Si), TiN/LaO/SiO_2_/Si, and TiN/ZrO/SiO_2_/Si are shown in Fig. 4(g,h and i), respectively.

Accordingly, we found the formation of interface dipole in TiN/(reference, LaO and ZrO)/SiO_2_/Si stack structure. In this case, La and Zr atoms are diffused into the interface of TiN/(LaO or ZrO) and (LaO or ZrO)/SiO_2_ by thermal annealing. For the first interface dipole, that is, the interface dipole is formed between (La doped- TiN, TiN) and (LaSiOx, ZrSiOx), and in the second interface dipole is formed between (LaSiOx, ZrSiOx) and SiOx layer. The second interface dipole can be attributed to the fact that oxygen is reduced at the interface between LaO and ZrO and the La(Zr) -O-Si bonding is transformed into La(Zr) -Si bonding at the interface^18^ by reducing the interfacial oxygen density. That is, interface dipole is formed by the change of La, Zr and oxygen in the interface.

In addition, Fig. 4 shows the work function depth profile where the vacuum level increases in the depth direction of TiN/(ref, ZrO, LaO)/SiO_2_. These results show the direction of the dipole moments. And these results show that the work function at the interface between TiN and oxide layers increases in the direction of depth. In other words, the vacuum level increases in the direction of depth in the TiN/(reference, LaO and ZrO)/SiO_2_ thin films. Further, we know the direction of the dipole moment. As shown in the Fig. 4(g,h and i), the direction of the interface dipole in both TiN/LaO and TiN/ZrO was the same.

Furthermore, in all groups, the direction of the SiO_2_/Si interface dipole was also the same, i.e., the dipole direction is opposite to the TiN/LaO and ZrO thin films. These results are good agreement with other report^19^. As a result, the EWF in ZrO shifted positively owing to the interface dipole. On the other hand, in case of LaO, the EWF was determined using the sum of the dipole and the La doped TiN effect, which reduces the EWF as described in Eq. (2).

## Conclusions

We developed techniques, which can be used to alter *V*_*FB*_ in high reliability devices with a TiN/SiO_2_ structure, and proposed driving mechanisms. By doping with La and Zr in TiN, *V*_*th*_ was controlled for both TiN/LaO and TiN/ZrO in high reliability devices. We suggested a semi-empirical equation that predicted the EWF and dipole direction as a function of composition ratio in each region of a TiN/LaO/SiO_2_/Si stack. Moreover, we confirmed that the EWF is measurable directly via the work function depth profiling by using AES system. In summary, this study presented a useful analytical method for directly determining the work function depth profiling, which can be useful to improve device characteristics including EWF, *V*_*FB*_, and *V*_*th*_.

## Additional Information

**How to cite this article:** Heo, S. *et al*. Direct evidence of flat band voltage shift for TiN/LaO or ZrO/SiO_2_ stack structure via work function depth profiling. *Sci. Rep.* **7**, 43561; doi: 10.1038/srep43561 (2017).

**Publisher's note:** Springer Nature remains neutral with regard to jurisdictional claims in published maps and institutional affiliations.

## Figures and Tables

**Table 1:** RBS composition ratio of top layer (La doped TiN) and LaO/SiO_2_ interface layer in the TiN/LaO/SiO_2_/Si structure.

Sample	Top layer (La doped TiN)				LaO/SiO_2_ interface layer (La silicate)		
	TiN	N	La	O	La	O	Si
LaO 4.5 Å	36.4	25.7	3.1	31.3	1.3	59.8	26.6
LaO 7 Å	21.2	12.3	5.0	54.7	1.5	61.4	25.0
LaO 10 Å	23.2	13.5	6.8	51.3	1.8	64.8	23.5

**Table 2:** RBS composition ratio of top layer (TiN) and ZrO/SiO_2_ interface layer in the TiN/ZrO/SiO_2_/Si structure.

Sample	Top layer (TiN)				ZrO/SiO_2_ interface layer (Zr silicate)		
	Ti	N	Zr	O	Zr	O	Si
ZrO 5 Å	34.4	45.6	0	20	5.7	65.8	20.3
ZrO 8 Å	34.4	45.6	0	20	11.2	63.7	17.2
ZrO 11 Å	34.4	45.6	0	20	18.8	58.5	17.3

## References

[b1] Colinge J. P. FinFETs and other multi-gate transistors. Springer (2008).

[b2] Leroux C. . Investigating doping effects on high-kappa metal gate stack for effective work function engineering. Solid State Electronics 88, 21–26 (2013).

[b3] Kadoshima M. . Effective-Work-Function Control by Varying the TiN Thickness in Poly-Si/TiN Gate Electrodes for Scaled High- k CMOSFETs. IEEE Electron Device Letters 30, 466 (2009).

[b4] Freeouf J. L. & Woodall J. M. Schottky barriers: An effective work function model. Appl. Phys. Lett. 39, 727 (1981).

[b5] Essa Z. . Evaluation and modeling of lanthanum diffusion in TiN/La2O3/HfSiON/SiO2/Si high-k stacks. Appl. Phys. Lett. 101, 182901 (2012).

[b6] Zheng X. H. . Diffusion behavior of dual capping layers in TiN/LaN/AlN/HfSiOx/Si stack. Appl. Phys. Lett. 99, 131914 (2011).

[b7] Bersch E. . Characterization of HfO2 and Hafnium Silicate Films on SiO2/Si. J. Appl. Phys. 108, 114107 (2010).

[b8] Spessot A., Caillat C., Fazan P., Ritzenthaler R. & Schram T. Understanding workfunction tuning in HKMG by Lanthanum diffusion combining simulations and measurements. *Proceedings of the International Conference on Simulation of Semiconductor Process and Devices*. IEEE., doi: 10.1109/SISPAD.2013.6650587 (2013, 09 03-05).

[b9] Gao M. & Brillson L. J. Application of high spatial resolution scanning work function spectroscopy to semiconductor surfaces and interfaces. J. Vac. Sci. Technol. B. 25, 334 (2007).

[b10] Yoshitake M. & Yoshihara K. Measurement of work function change with surface segregation of substrate element on a deposited film. Appl. Surf. Sci. 146, 97 (1999).

[b11] Boujamaa R. . Impact of high temperature annealing on La diffusion and flatband voltage (Vfb) modulation in TiN/LaOx/HfSiON/SiON/Si gate stacks. J. Appl. Phys. 111, 054110 (2012).

[b12] Lee D. . Effective work function engineering for a TiN/XO(X = La, Zr, Al)/SiO2 stack structures. Appl. Phys. Lett. 108, 212102 (2016).

[b13] Copel M. Selective desorption of interfacial SiO2. Appl. Phys. Lett. 82, 1580 (2003).

[b14] Ming Z. . Si emission from the SiO2∕Si interface during the growth of SiO2 in the HfO2∕SiO2∕Si structure. Appl. Phys. Lett. 88, 153516 (2006).

[b15] Watanabe H. Roughness at ZrO2/Si interfaces induced by accelerated oxidation due to the metal oxide overlayer. Appl. Phys. Lett. 83, 4175 (2003).

[b16] Kageshima H., Shiraishi K. & Uematsu M. Universal Theory of Si Oxidation Rate and Importance of Interfacial Si Emission. Jpn. J. Appl. Phys. 38, L971 (1999).

[b17] Sakai Y., Kudo M. & Nielsen C. Surface potential measurement with high spatial resolution using a scanning Auger electron microscope. J. Vac. Sci. Technol. A 19, 1139 (2001).

[b18] Yang Z. C. . Role of interface dipole in metal gate/high-k effective work function modulation by aluminum incorporation. Appl. Phys. Lett. 94, 252905 (2009).

[b19] Huang A. P. . Flat-band voltage shift in metal-gate/high-k/Si stacks. Chinese Phys. B. 20, 097303 (2011).

